# Role of the Accessory Parotid Gland in the Etiology of Parotitis: Statistical Analysis of Sialographic Features

**DOI:** 10.1371/journal.pone.0150212

**Published:** 2016-02-25

**Authors:** Wangyong Zhu, Fengchun Hu, Xingguang Liu, Songcan Guo, Qian Tao

**Affiliations:** 1 Department of Oral Maxillofacial-Head and Neck Oncology, the Affiliated Hospital of Stomatology, Sun Yat-sen University, Guangzhou, 510055, China; 2 Guangdong Provincial Key Laboratory of Stomatology, Sun Yat-sen University, Guangzhou, Guangdong, 510055, China; 3 School of Engneering, Sun Yat-sen University, Guangzhou, 510275, China; School of Medicine and Health Sciences, University of North Dakota, UNITED STATES

## Abstract

This retrospective study aimed to identify if the existence of the accessory parotid gland correlated with the etiology of parotitis. This may aid the development of better treatment strategies in the future. Sialographic features of cases with parotitis and healthy subjects were reviewed. The chi-square test was used to compare the incidence of accessory parotid gland between the groups. The Student’s t test was used to compare the length of Stensen’s duct, the length from the orifice to the confluence of the accessory duct, and the angle between the accessory duct and Stensen’s duct between the groups. The incidence of accessory parotid gland in patients with parotitis was 71.8% (28/39), which was significantly higher than that in healthy subjects (P = 0.005). Patients with parotitis had a longer Stensen’s duct than healthy subjects (P = 0.003). There was no significant difference in the length from the orifice to the confluence of the accessory duct or the angle between the accessory duct and Stensen’s duct (P = 0.136 and 0.511, respectively) between the groups.

The accessory parotid gland might play a role in the pathogenesis of parotitis. The existence of an accessory parotid gland is likely to interfere with salivary flow. Computational fluid dynamics analysis of salivary flow in the ductal system would be useful in future etiologic studies on parotitis.

## Introduction

Chronic parotitis is characterized by recurrent pain and swelling in the affected parotid gland. The symptoms of parotitis are often intermittent and mild and may be related to eating in the early stages of disease. Its etiology is considered to be multifactorial, and its exact pathogenesis is not fully understood. Obstructive causes of reduced salivary flow include sialoliths, stricture of the main duct or orifice, mucus plugging, external pressure on the duct, and developmental malformations of the duct [1–4]. The presence of an accessory parotid gland (APG) is a fairly common anatomical variation. The incidence of APG ranges between 21 and 56% [5,6]. The APG is a small gland with a diameter of 0.5–1 cm, and is located on average 6 mm anterior to and separate from the parotid gland. In most cases, it drains into Stensen’s duct (SD) through an accessory duct (AD). Horsburgh et al. found that 59% of patients with parotitis had an APG, which is a higher rate than that observed in healthy people [7]. This suggests that the presence of an APG might be related to the pathogenesis of parotitis.

The current study aimed to explore the role of the APG in the etiology of parotitis. We analyzed the incidence of APG and the anatomical parameters of the parotid gland between healthy subjects and patients with parotitis.

## Materials and Methods

This study was approved by the institutional ethics board of the Hospital of Stomatology, Sun Yat-sen University. All patients were informed of the details of the study and informed consent was obtained. Patient information was de-identified prior to analysis.

All procedures were performed by one doctor using the same standard technique: 2 mL of 40% iodinated oil (a contrast medium) was gently introduced into the orifice of the parotid duct, until the patient felt an obvious sense of swelling in the gland. The maxillofacial region was scanned transversely and sagittally using a cone-beam computed tomography scanner. The following imaging parameters were used: 108 mA; 90 kV; field of vision, 500 × 500; slice thickness, 0.4 mm.

The main part of each patient’s parotid was reconstructed and modeled by Mimics 14.11 software (Materialise Technologies, Leuven, Belgium). Independently, two experienced radiologists retrospectively reviewed all anonymous images demonstrating the entire ductal system. The authors reviewed the data and used electronic calipers provided by the Mimics 14.11 software to measure the following parameters: the length of SD, the length between the confluence of the AD and the orifice, and the angle between the AD and SD.

The inclusion criteria of the parotitis group were: 1. The patient was diagnosed as having parotitis; 2. The radiologists could analyze the sialographic features of the images that demonstrated the entire ductal system. The inclusion criteria of the healthy subjects group were: 1. The volunteer had no history of salivary gland disease; 2. The radiologists could analyze the sialographic features of the images that demonstrated the full extent of the ductal system.

Statistical analysis was performed using SPSS version 15.01 software (SPSS, Inc., Chicago, IL). The chi-square test was used to compare the difference in APG incidence between the healthy and parotitis groups. The Student’s t test was used to compare the sialographic features of the two groups, with the significance level set at P < 0.05.

## Results

Seven males and 11 females with a mean age of 22.5 years old were included in the healthy subjects group. Based on 31 sets of sialographic data from 15 left parotid and 16 right parotid glands, 38.7% of cases had APGs. In one case, the APG drained into SD through two ADs. S1 Fig shows typical images from the healthy subjects group.

Twelve males and 19 females with a mean age of 44.3 years old were included in the parotitis group. Based on 39 sets of sialographic data from 19 left parotid and 20 right parotid glands, 71.8% of cases had APGs. Retrospective evaluation of the images revealed that 23 of 39 (59.0%) cases exhibited sialographic features of parotitis. Seventeen cases presented with punctiform or globular dilatation, 7 presented with a filling defect in SD, and 10 presented with irregular duct segmental dilatation and narrowing. In two cases, the APG drained into SD through two ADs. S2 Fig shows typical images from the parotitis group. Remarkably, the characteristics of some patients in this group showed correlation with the APD upon sialography. In the typical case shown in S3 Fig, the patient presented with swelling and pain in the parotid region during eating. The upstream SD was dilated and the downstream duct was normal, bounded by the confluence of the AD with SD. By using sialendoscopy, we observed mucous embolus in the duct, but did not find any sialoliths or stenosis.

The incidence of APG and the length of SD in patients with parotitis were significantly higher and longer than those in healthy subjects (P = 0.005 and 0.003, respectively). There was no significant difference in the length from the orifice to the confluence of the AD (P = 0.136) or the angle between the AD and SD (P = 0.511) between the healthy and parotitis groups. Table 1 shows the results in detail as means, standard deviations, and ranges.

**Table 1:** Comparison of Anatomical Parameters of the Parotid Gland Between Two Groups.

	Healthy group (n = 31)				Parotitis group (n = 39)				P
	n	Mean	Standard deviation	Range	n	Mean	Standard deviation	Range	
Accessory parotid gland	12				28				0.005
Length of SD (mm)	31	51.7	8.8	36.1–82.9	39	58.0	8.3	39.7–76.1	0.003
Length from orifice to confluence of AD (mm)	12	30.0	5.1	19.9–39.9	28	33.3	8.1	14.3–45.2	0.136
Angle between AD and SD (°)	13	45.7	12.0	24.7–58.3	30	42.6	15.1	20.3–79.6	0.511

SD, Stenson’s duct; AD, accessory duct

## Discussion

The exact pathogenesis of parotitis is still not completely understood. Reduced salivary flow is likely to be the most important factor, since it causes the repeated appearance of ascending infection in the parotid gland. This leads to further destruction of the acini and an increase in the mucous substances of the saliva, which aggravates the disease [1–4]. The obstructive causes of swelling in the parotid gland are examined by sialendoscopy, and include sialoliths (20.3–52.2%), stenosis of the duct, foreign bodies, and fibrous embolus (32.6–56.3%). However, obstructive causes are only reported to be found in 53.5–89.3% of cases [8,9]. This is quite similar to our clinical experience of parotitis. It is often hard to explain the cause of symptoms because nearly half of patients with parotitis do not have any sialoliths or stenosis in their ducts. It is known that there are complex rheological forms of salivary flow such as flow at the duct entrance, secondary flow, spiral flow, flow separation, and recirculation at the turnings and confluences of the ducts [10]. If we regard saliva flow in the ductal system as a micro flow field, changes of fluid mechanics in the ductal system of the parotid might explain the reduced salivary flow observed in patients without obstruction.

The APG is an anatomical variant with an incidence of 21–56% [5,6]. However, Horsburgh et al. found that the presence of an APG is more common in patients with parotitis, with an incidence of 59% [7]. Therefore, we focused on the role of the APG in the etiology of parotitis. In our study, we found the incidence of APG in patients with parotitis to be 71.8%, which was significantly higher than the incidence of APG in healthy subjects. Thus, we speculate that the high incidence of APG correlates to the pathogenesis of parotitis. Perhaps the existence of an APG increases the risk of parotitis. It may be possible that the extra gland and confluence contribute to the development of sialoliths or mucus embolus, which in turn could lead to reduced salivary flow.

The component of saliva produced by the APG might differ from that produced by the parotid gland. Previous studies report that 71–90% of cases of sialolithiasis occur in the submandibular gland, while 5–29% occur in the parotid [11,12]. The parotid is a serous gland and the saliva that it produces is less likely to develop sialoliths due to its low viscosity in comparison with the saliva produced by the submandibular gland. Previous reports have measured the viscosity of saliva produced by each salivary gland. The zero-shear-rate viscosity of saliva in the submandibular gland is 3.1–10 times that of the parotid gland [13,14]. Toh et al. found that 26.7% (8/30) of APGs contain both serous and mucous acini [5]. As it is produced by mixed acinus, the saliva of the APG is more sticky, which is more likely to contribute to parotitis. There is a case report of a female with sialadenitis in the APG. After total parotidectomy was carried out, the patient complained of pain and swelling as before. Finally, it was found that mucoid saliva was being produced by the APG [15]. Furthermore, this kind of saliva might facilitate the development of sialoliths in the confluence of the AD and SD. Cases of sialolithiasis occurring in the APG have been reported, which highlights the possibility of sialoliths developing in the APG [16–18]. This might suggest a pathophysiological reason as to why the presence of an APG correlates to the pathogenesis of parotitis.

Abnormal morphology of the salivary duct can interfere with the flow of saliva. An anatomical malformation has been discovered in the submandibular hilus, known as a pelvis-like formation. In this anatomical variation, the bifurcation or trifurcation of the hilus is replaced by a basin-like structure. The accumulation and convergence of saliva in this pelvis-like formation causes obstruction and even lithogenesis [9]. Moreover, in 1991, Katz et al. were the first to describe the observation of smooth muscle strands around the walls of Wharton’s duct, which are thought to have a sphincter-like mechanism. It is considered possible that this anatomical malformation might play a role in the process of lithogenesis [19]. Subsequently, Nahlieli et al. also observed and documented this anatomical malformation in SD, which is located posteriorly, in the vicinity of the ramification [9]. In our study, there was no difference in APG morphology between the two groups, including the location of and angle between the AD and SD. Therefore, we suspect that the confluence itself might lead to disturbance of salivary flow. In the typical case shown in S3 Fig, the patient presented with swelling and pain in the parotid without sialoliths or stenosis as determined by sialendoscopy. As shown in S3 Fig, the sialographic features of the duct were separated by the confluence. It appeared that the dilated upstream section of the duct was blocked in the region of the confluence, although there were no sialoliths or stenosis present.

Changes in salivary flow occur essentially either because of changes in saliva composition or differences in ductal morphology. Computational fluid dynamics analysis of salivary flow in the ductal system would be a useful future direction in etiologic studies of parotitis. At present, computational fluid dynamics analysis is commonly applied in the study of blood flow in the cardiovascular system and air flow in the respiratory tract, but is rarely used in the study of salivary flow. Zhu et al. explored the effect of salivary flow on sialolithogenesis using computational fluid dynamics analysis. They demonstrated a low velocity zone and a vortex zone forming around sialoliths, which exacerbated their development [20]. We speculate that if there is a special flow field in the confluence of the AD with SD, this could block salivary flow and led to the increased risk of parotitis. This might explain the reduced salivary flow observed in patients without obstruction.

The APG might be related to the pathogenesis of parotitis. It remains uncertain whether the existence of this anatomical variant increases the risk of parotitis. More histopathological, hydromechanical, and clinical studies of the APG are needed to support this hypothesis.

## Supporting Information

S1 Fig Typical cases from the healthy subjects group. (A) No accessory parotid gland (APD) present. (B) One APD present (arrowhead). (C) One APD with two accessory ducts (ADs) present (arrowhead).
(JPG)

S2 Fig Typical cases from the parotitis group. (A) No accessory parotid gland (APD) present. Patient diagnosed with irregular duct segmental sialectasis (arrow). (B) One APD present (arrowhead) in a case with punctiform or globular dilatation (arrows). (C) One APD with two accessory ducts (ADs) present (arrowhead).
(JPG)

S3 Fig Example of a typical case. The upstream Stenson’s duct (SD) was dilated and the downstream duct was normal, bounded by the confluence of the accessory duct (AD) with SD (arrowhead).
(JPG)
