# Supplementary figures and images for: Role of the Accessory Parotid Gland in the Etiology of Parotitis: Statistical Analysis of Sialographic Features
Source: PLoS One. 2016 Feb 25;11(2):e0150212. doi: 10.1371/journal.pone.0150212 (PMC4767724; doi:10.1371/journal.pone.0150212)

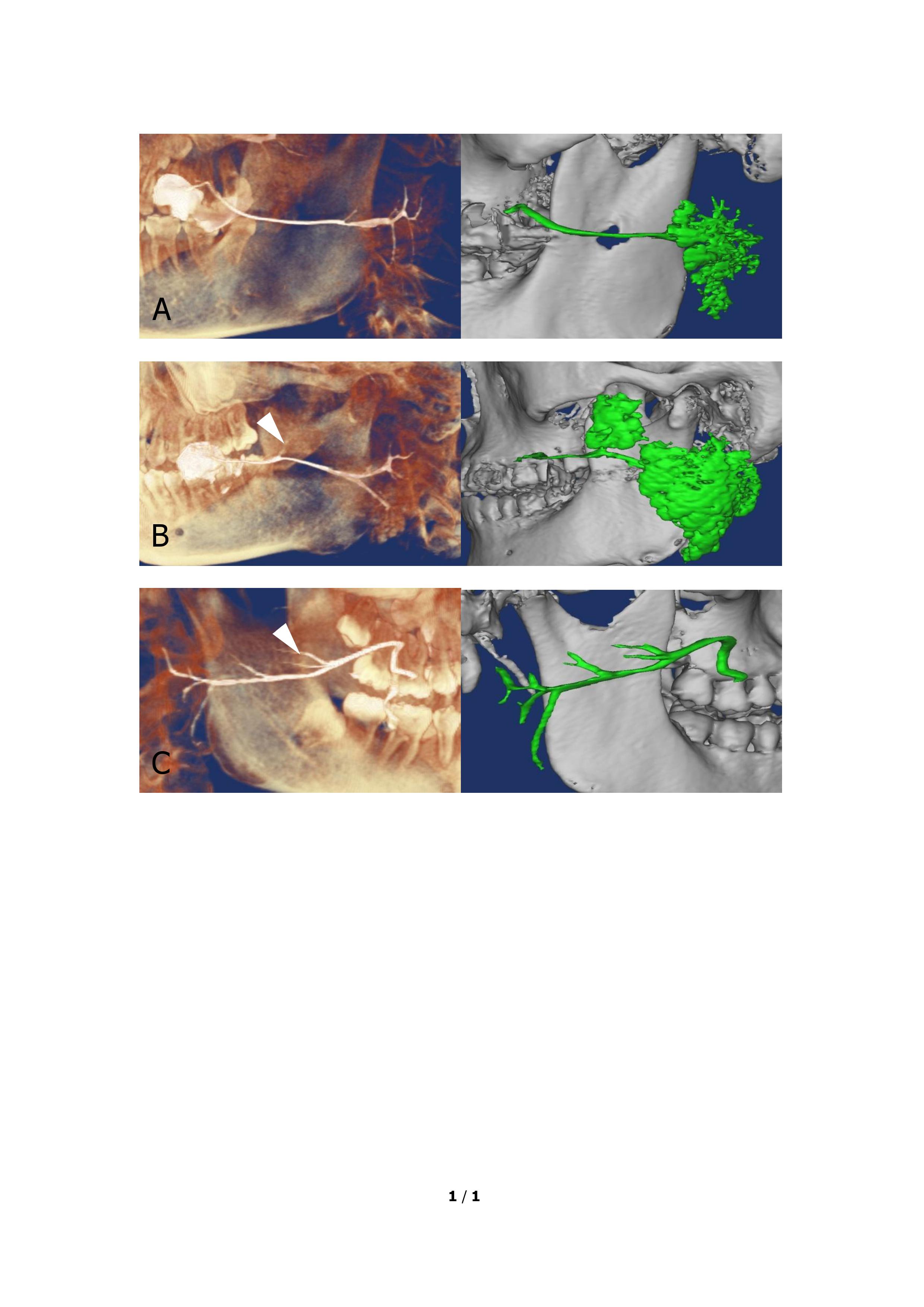

Supplement: S1 Fig — (A) No accessory parotid gland (APD) present. (B) One APD present (arrowhead). (C) One APD with two accessory ducts (ADs) present (arrowhead). (JPG) [file pone.0150212.s001.jpg]

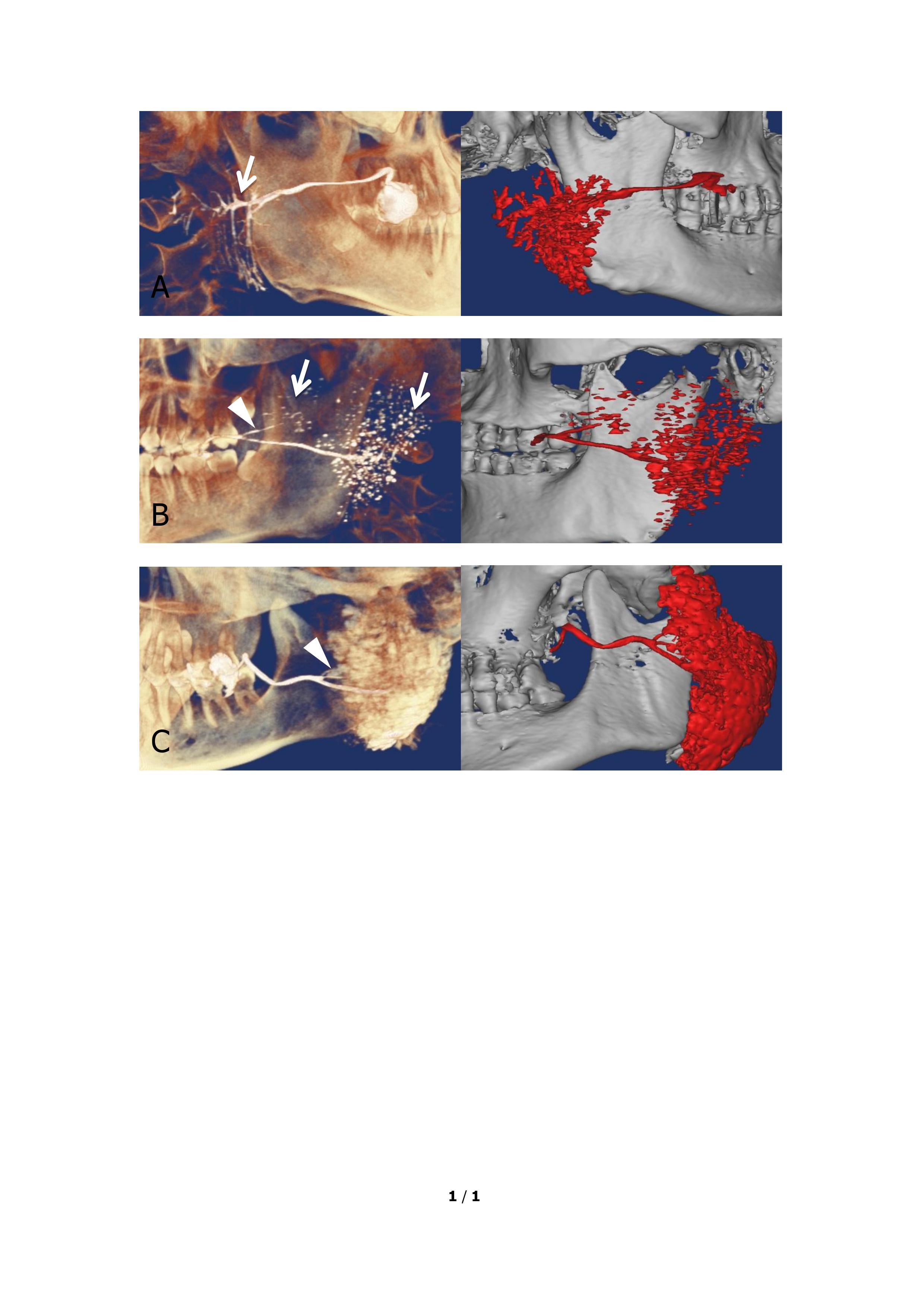

Supplement: S2 Fig — (A) No accessory parotid gland (APD) present. Patient diagnosed with irregular duct segmental sialectasis (arrow). (B) One APD present (arrowhead) in a case with punctiform or globular dilatation (arrows). (C) One APD with two accessory ducts (ADs) present (arrowhead). (JPG) [file pone.0150212.s002.jpg]

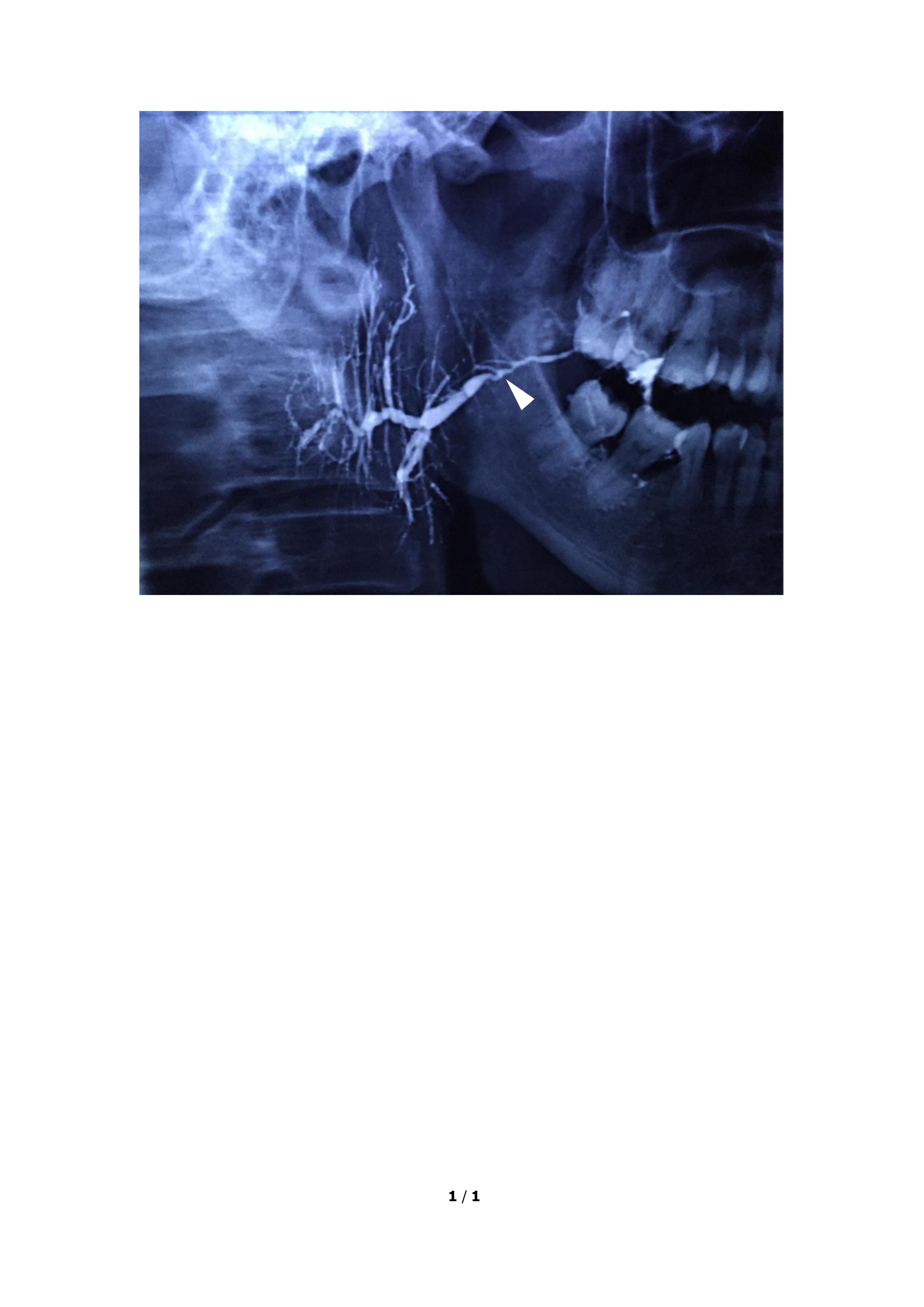

Supplement: S3 Fig — The upstream Stenson’s duct (SD) was dilated and the downstream duct was normal, bounded by the confluence of the accessory duct (AD) with SD (arrowhead). (JPG) [file pone.0150212.s003.jpg]
